# HIV diagnosis and treatment status in individuals dying from HIV in Zimbabwe, Malawi, and South Africa: A model comparison analysis

**DOI:** 10.1371/journal.pmed.1004864

**Published:** 2026-09-10

**Authors:** Loveleen Bansi-Matharu, Daniel T. Citron, Rowan Martin-Hughes, Haroon Moolla, John Stover, Michael Pickles, Tara Mangal, Jenny Smith, Owen Mugurungi, Mmatlou Simeon Kubyana, Isaac Taramusi, Valentina Cambiano, Amon Mpofu, Debra ten Brink, Edinah Mudimu, Tsitsi Apollo, Anna Bershteyn, Linley Chewere, Dobromir Dimitrov, Stephen Macheso, Leigh F. Johnson, Andrew Phillips

**Affiliations:** 1 Institute for Global Health, UCL, London, United Kingdom; 2 Department of Population Health, New York University Grossman School of Medicine, New York, New York, United States of America; 3 Burnet Institute, Melbourne, Australia; 4 Centre for Integrated Data and Epidemiological Research, University of Cape Town, Cape Town, South Africa; 5 Avenir Health, Takoma Park, Maryland, United States of America; 6 MRC Centre for Global Infectious Disease Analysis, Jameel Institute, School of Public Health, Imperial College London, London, United Kingdom; 7 Centre for Health Economics, University of York, York, United Kingdom; 8 Ministry of Health & Child Welfare, Harare, Zimbabwe; 9 Department of Decision Sciences, University of South Africa, Pretoria, South Africa; 10 UNAIDS UCO, Harare, Zimbabwe; 11 National AIDS Council, Harare, Zimbabwe; 12 Ministry of Health and Child Care, HIV Program, Harare, Zimbabwe; 13 Department of HIV, STI & Hepatitis, Ministry of Health, Lilongwe, Malawi; 14 Public Health Sciences Division, Fred Hutchinson Cancer Center, Seattle, Washington, United States of America; 15 UNICEF Country Office, Lilongwe, Malawi (SM); University of Glasgow, UNITED KINGDOM OF GREAT BRITAIN AND NORTHERN IRELAND

## Abstract

**Background:**

Antiretroviral therapy (ART) has greatly reduced HIV-related mortality and improved life expectancy in sub-Saharan Africa since the early 2000s. However, HIV-related mortality remains high. To guide intervention priorities, we used seven established HIV simulation models to identify where in the HIV care cascade deaths occur in Zimbabwe, Malawi, and South Africa. This multi-model approach provided a more comprehensive and robust assessment than could be achieved using any single modelling approach.

**Methods and findings:**

To assess alignment, each model generated key HIV metrics from 2000 to 2045, including total population, HIV prevalence, annual new infections, and proportions of people with HIV (PWH) who were diagnosed, on ART, and virally suppressed. HIV-related deaths were estimated annually and categorised by whether they occurred in individuals who were (1) undiagnosed, (2) diagnosed but had not yet started ART, (3) on ART, or (4) had interrupted ART.

The models showed strong consistency with population estimates and HIV prevalence across the different settings. There was, however, variation in the absolute number of HIV-related deaths between models; in Zimbabwe in 2025, this ranged from 3,666 to 17,475; in Malawi from 4,580 to 17,835; and in South Africa from 39,470 to 114,968. In Zimbabwe, all models consistently indicated that PWH receiving ART made up the largest proportion of HIV-related deaths, though this proportion differed across models, ranging from 35% to 64%. This was followed by HIV-related deaths amongst those who had interrupted ART, ranging from 17% to 29%. Similar trends were seen in Malawi. In South Africa, a high proportion of HIV-related deaths occurred amongst people who had interrupted ART (23% to 74% in 2025) as well as those who were currently receiving ART (23% to 56%). Models are reliant on empirical data and limited data availability—in this instance, lack of national death registries—is a key constraint on modelling studies.

**Conclusion:**

Absolute numbers of HIV-related deaths vary substantially between models, highlighting wider issues around ascertainment of death in the region. However, our results do consistently show that most deaths are occurring amongst PWH on ART in Zimbabwe and Malawi, and amongst those who have interrupted treatment in South Africa, suggesting interventions around adherence counselling and retention in care need to be prioritised.

## Author summary

**Why was this study done?:**

- Antiretroviral therapy (ART) has substantially reduced HIV-related deaths and improved life expectancy in sub-Saharan Africa, but HIV-related mortality remains high.
- Understanding where HIV-related deaths occur along the HIV care cascade (from diagnosis to treatment and retention) can help prioritise interventions.
- We used multiple established HIV simulation models to identify where in the HIV care cascade HIV-related deaths occur in Zimbabwe, Malawi, and South Africa. Using several different models gave us a more complete and reliable picture than relying on a single model alone.

**What did the researchers do and find?:**

- Seven HIV simulation models contributed to this analysis, and to assess alignment between models, epidemic trends including HIV prevalence, new infections, and engagement in care (diagnosis, ART use, and viral suppression) were analysed.
- HIV-related deaths were categorised by care status: undiagnosed, diagnosed but not on ART, on ART, or having interrupted ART.
- The models showed strong agreement on population size and HIV prevalence, but varied widely in the estimated number of HIV-related deaths. Despite this variation, consistent patterns emerged. In Zimbabwe and Malawi, most HIV-related deaths occurred among people receiving ART, followed by those who had interrupted ART while in South Africa, a large share of deaths occurred among those who had interrupted ART, alongside those currently on ART.

**What do these findings mean?:**

- Differences in estimated death numbers across models highlight uncertainty in measuring HIV-related mortality, and general all-cause mortality, in the region.
- However, consistent patterns suggest that many deaths occur among people already engaged in care, particularly those on ART or those who have interrupted treatment.
- Interventions to improve adherence and retention in care need to be prioritised to further reduce HIV-related mortality.
- One limitation of this study is the lack of national death registries in the region; models are reliant on empirical data and limited data availability is a key constraint on modelling studies, resulting in wide variation in HIV mortality estimates. Secondly, these analyses were conducted before the recent funding USAID cuts and our results may be conservative since the situation following the cuts may be even more concerning.

## Abstract 3

In a comparison of seven HIV simulation models, Loveleen Bansi-Matharu and colleagues identify distinct points in the HIV care cascade where deaths occur in people with HIV in Zimbabwe, Malawi and South Africa, suggesting adherence and retention in care are key targets for intervention.

## Introduction

Antiretroviral therapy (ART) has had a huge impact on HIV mortality rates worldwide and specifically in generalised HIV epidemic settings in sub-Saharan Africa. Since the provision of ART was scaled up gradually from 2003 onwards, virological and immunological outcomes have dramatically improved and life expectancy for adults with HIV has increased substantially [1,2].

In Africa, only a third of all deaths are officially recorded in a national registry and only a third of countries in the region record and report annual death numbers (of which only 4 countries (including South Africa) have records that meet international standards for documenting cause of death) [3]. While surveillance data is limited in the region, in eastern and southern Africa, death rates amongst people with HIV (PWH) have been reduced by half between 1990–2003 and 2009–2011 due to expansion of ART [4]. Estimates from countries within the region similarly indicate reductions in mortality rates among different HIV populations. For example, in South Africa between 2000 and 2014, the rollout of ART was estimated to have reduced the cumulative number of adult HIV-related deaths by 1.72 million [5]. In Rwanda, an increase in life expectancy between 1997–2007 and 2008–2011 was reported, from 20 additional years to 26 additional years for a PWH aged 20 years [6], and in South Africa, life expectancy for PWH was reported to be near normal for those initiating ART with CD4 counts >200/mm^3^ [7]. More recent estimates from UNAIDS show that average life expectancy in the region has increased from 56 years in 2010 to 61 years in 2023 [2].

Despite the lower death rates amongst people with HIV over the last two decades, HIV-related mortality remains a major issue in Eastern and Southern Africa where 260,000 deaths were attributable to HIV in 2023 (UNAIDS Regional profile [8]). Previous evidence has shown that death rates among PWH were significantly higher for those not on ART or those who began treatment with low CD4 counts [5,9]. Since then, ART coverage has continued to expand across most settings in sub-Saharan Africa and remains essential in closing the gap between mortality rates amongst those with HIV and those without. However, it is important to identify the stages of HIV care at which deaths take place in order to inform priorities for HIV interventions, i.e., amongst those undiagnosed, diagnosed without initiating ART, on ART or having interrupted ART. This is of particular importance in the current funding landscape where HIV programmes are facing devastating cuts, which may potentially reverse the gains made over the last two decades [10–12].

Previously published studies from the HIV Modelling Consortium have focussed on Zimbabwe, Malawi and South Africa where HIV epidemiology and programmes differ; recent modelled data show in Zimbabwe, 95% of PWH are diagnosed, 95% are on ART and 91% are virally suppressed [13]. In Malawi, these metrics are 95%, 90% and 86% respectively, while in South Africa these are estimated at 95%, 81% and 74%, respectively. Building on previous work of the HIV Modelling Consortium [14,15], we present outputs from several well-established HIV simulation models to identify at which stage of the HIV care cascade deaths amongst those ages 15+ are occurring in Zimbabwe, Malawi and South Africa, how this has changed over time and how it would be predicted to change if programmes continue much as they are over the foreseeable future. Seven independent modelling groups contributed outputs for this analysis (a maximum of five per country), providing a comprehensive and robust assessment than could be achieved with any single modelling approach.

## Methods

The seven models contributing to this analysis—Optima HIV, EMOD, Goals, Thembisa, PopART-IBM, HIV Synthesis and Thanzi La Onse (TLO) —have been previously described [16–22]. Each model was independently parameterised and calibrated to one or more of the settings modelled: Zimbabwe (Goals, Optima HIV, HIV Synthesis, PopART-IBM and EMOD), Malawi (EMOD, Optima HIV, TLO and HIV Synthesis) and South Africa (EMOD, Optima HIV, HIV Synthesis and Thembisa). These three countries are those involved in the Modelling to Inform HIV Programmes in sub-Saharan Africa (MIHPSA) project (http://hivmodeling.org/mihpsa) led by the HIV Modelling Consortium [23,24].

Key model features are summarised in Table A in S1 Appendix, including how HIV deaths were determined for each model. Models contributing outputs to these analyses were a mixture of compartmental and individual-based models. Each model was constructed and parameterised using different structures and different underlying assumptions; this enabled us to ensure that outputs reflected a wide range of modelling approaches and hence there was no attempt to align on model parameterisation or assumptions for the purpose of this exercise. The analyses were conducted in 2024, before the USAID funding cuts. Modelling groups were asked to assume the epidemic would continue as it would have in the absence of the funding cuts, with programmes proceeding broadly as before. Assumptions on the future epidemic are shown in Table A in S1 Appendix.

HIV-related deaths among individuals aged 15 years and older were defined as deaths directly or indirectly attributable to HIV. These were informed by estimates of excess HIV mortality, under the assumption that HIV mortality rates are equal to the excess mortality rate when compared against the HIV–negative population. The modelling approach for each model was as follows: In HIV Synthesis, an underlying base rate of advanced HIV disease dependent on current CD4 count, viral load, age and being on ART was further multiplied by a factor dependent on occurrence of TB/AIDS defining conditions. This final rate was used to determine the probability of HIV-related deaths [25,26] (https://doi.org/10.6084/m9.figshare.32895719). In Optima HIV, HIV-related mortality rates were determined by population-based estimates on current CD4 count, treatment status, and viral suppression status for those who are treated, with a cofactor for HIV mortality rate based on tuberculosis prevalence. Detailed sources for HIV-related mortality rates are specified in the Optima HIV User Guide (available via https://optimamodel.com/hiv/). In EMOD, HIV-related mortality rates were determined for each simulated individual according to their age, sex, current CD4 count, ART status and time since most recently initiating ART (https://doi.org/10.5281/zenodo.21242723; https://github.com/BershteynLab/MWI_MIHPSA/releases/tag/MWIMIHPSA; https://github.com/Edinah-Research-Lab/ZA_HIV_Deaths_And_Transmissions) [27]. In Goals, HIV-related mortality rates among PLHIV not on ART are defined by CD4 count and age based on fitting to data from the ALPHA Network and PHIAs [28]. For PLHIV who are on ART mortality rates are defined by sex, age, CD4 count at treatment initiation, duration on treatment and region for PLHIV on ART based on analysis of treatment cohort data [29] (https://avenirhealth.org/software-spectrum.html). In PopART-IBM, HIV-related mortality rates were determined for each simulated individual based on their current CD4 count and ART status, including whether they had initiated ART in the past six months or longer. In the TLO model, HIV-related mortality among adults is determined by age at seroconversion and time since infection, with survival times parameterised using the ALPHA Network pooled cohort analysis (https://zenodo.org/records/21207965, https://doi.org/10.5281/zenodo.21207965) [30]. In adults who initiate antiretroviral therapy, mortality is determined by viral-suppression status: sustained suppression is assumed to eliminate AIDS mortality, whereas individuals on ART without suppression retain the untreated hazard (The Thanzi La Onse Model | Zenodo). In Thembisa, HIV-related mortality rates in ART-naïve individuals are specified by age, sex and CD4 count, and are estimated by calibrating the model to South African vital registration data [5]. HIV-related mortality rates in ART-experienced individuals are specified by age, sex, baseline CD4 count and time since first ART initiation, and are estimated using data from the International epidemiology Databases to Evaluate AIDS (IeDEA) collaboration for Southern Africa [31] (http://dx.doi.org/10.2139/ssrn.7041598; https://github.com/leighjohnson/Thembisa.)

For each country, modelling groups produced key HIV metrics from 2000 to 2045 including the total population of the country, HIV prevalence, the number of new HIV infections annually, and the proportions of PWH diagnosed, on ART and virally suppressed.

For each model in each setting, the numbers and proportions of HIV-related deaths were generated from 2010 to 2040 for each year, stratified according to whether (1) the death occurred in a person who was undiagnosed *(a person living with HIV who had not yet been diagnosed by a healthcare professional)*, (2) was diagnosed but had not yet started ART *(a person diagnosed with HIV but had not started antiretroviral therapy)*, (3) was receiving ART *(a person diagnosed with HIV and receiving treatment)* or (4) had interrupted ART *(a person diagnosed with HIV who had started treatment and had subsequently interrupted treatment)*. Outputs from Goals were stratified according to whether the death occurred while on ART or not on ART and for Thembisa, estimates provided were lower bounds for those on ART and upper bounds for those who have interrupted. These initial categories were further broken down according to age and sex, time since start of ART, CD4 at start of ART and viral load at time of death (depending on whether models were able to record this information, see S1 Appendix).

For stochastic models, ensemble means were calculated across runs. Given the large volume of model outputs, uncertainty ranges were not presented beyond those inherent in our presentation of results for each model individually. Instead, we reported structural uncertainty across the range of estimates produced by different models.

This study was reported with consideration of the modelling reporting recommendations described by Garnett and colleagues for simulation-based modelling studies [32]

## Results

### Key HIV epidemic characteristics

**Fig 1:**
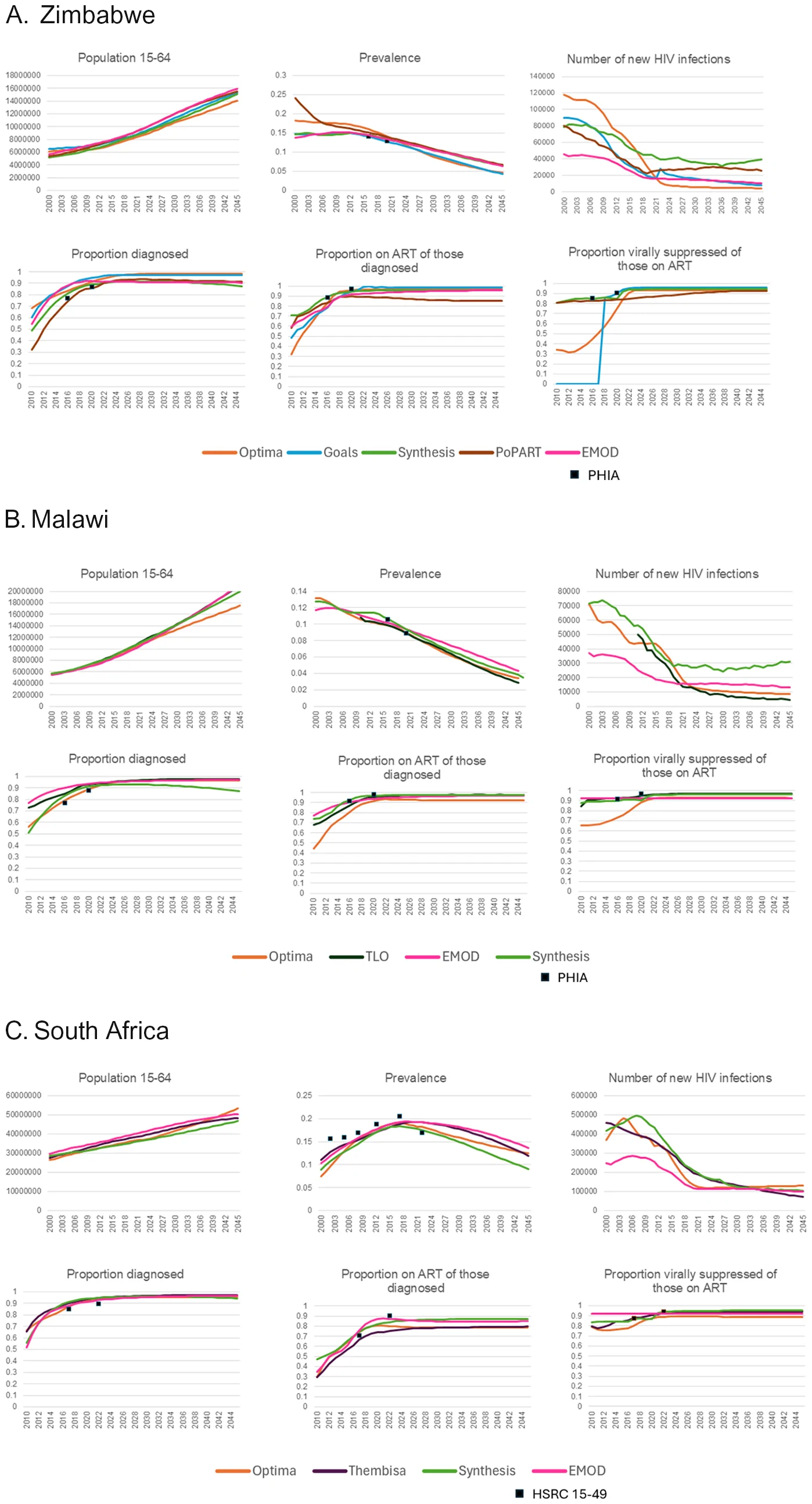
HIV epidemic characteristics for adults aged 15+ in Zimbabwe, Malawi and South Africa. x-axis: calendar year; y-axis specific to figure: Population: Number; Prevalence: proportion; Number of new HIV infections: Number; Proportion diagnosed, on ART and virally suppressed: proportion.

Fig 1A–1C summarise key HIV epidemic characteristics for Zimbabwe, Malawi, and South Africa.

Models aligned well on population estimates and HIV prevalence. Despite variation in predicted new infections, they generally agreed on the proportions of PWH diagnosed, on ART, and virally suppressed. In Zimbabwe, HIV Synthesis, EMOD, and PopART-IBM produced lower estimates of the proportion diagnosed than the other models, likely because they assumed that the number of HIV tests performed would remain constant despite population growth. PopART-IBM showed fewer diagnosed people on ART, driven mainly by higher assumed ART discontinuation rates. Models for Malawi were broadly consistent, showing trends similar to Zimbabwe, including variation in new infection estimates. For South Africa, new infection estimates were more consistent. Thembisa and HIV Synthesis did, however, project declining incidence over the next 20 years, while others indicated stable or slightly increasing annual HIV infections. These projections were in line with the assumption that the funding landscape would remain as it was in 2024.

### Absolute number of HIV-related deaths over time

**Fig 2:**
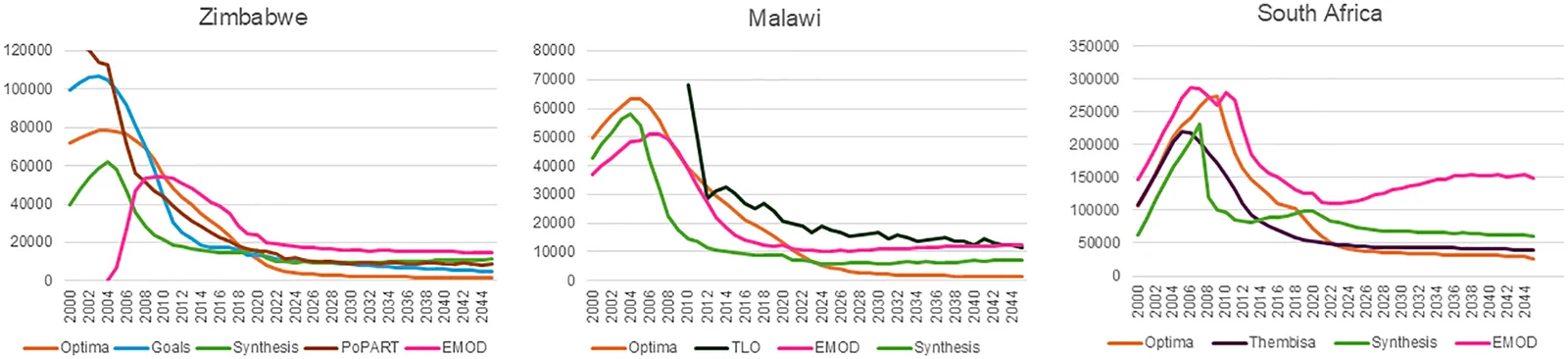
Absolute number of HIV-related deaths over time. x-axis: calendar year; y-axis: number.

The total number of HIV-related deaths over time is shown in Fig 2 and broken down by age in Table 1.

**Table 1:** Percentage of HIV-related deaths in 2025, stratified by age.

		15–34 years	35–49 years	50+ years	Total
Zimbabwe	Optima	40% (*n* = 1,479)	42% (*n* = 1,544)	18% (*n* = 643)	**3,666**
	Synthesis	13% (*n* = 1,240)	45% (*n* = 4,435)	42% (*n* = 4,169)	**9,844**
	Goals	27% (*n* = 2,784)	35% (*n* = 3,569)	38% (*n* = 3,828)	**10,181**
	PopART-IBM	20% (*n* = 2,104)	41% (*n* = 4,412)	39% (*n* = 4,189)	**10,705**
	EMOD	20% (*n* = 3,493)	48% (*n* = 8,389)	32% (*n* = 5,593)	**17,475**
Malawi	Optima	31% (*n* = 1,402)	43% (*n* = 1,976)	26% (*n* = 1,202)	**4,580**
	Synthesis	10% (*n* = 577)	38% (*n* = 2,263)	52% (*n* = 3,131)	**5,971**
	EMOD	17% (*n* = 1,741)	44% (*n* = 4,612)	39% (*n* = 4,039)	**10,392**
	TLO	35% (*n* = 6,220)	39% (*n* = 6,987)	26% (*n* = 4,628)	**17,835**
South Africa	Optima	37% (*n* = 14,665)	40% (*n* = 15,913)	23% (*n* = 8,892)	**39,470**
	Synthesis	11% (*n* = 8,255)	46% (*n* = 33,780)	43% (*n* = 31,644)	**73,679**
	EMOD	14% (*n* = 15,590)	53% (*n* = 60,951)	33% (*n* = 38,427)	**114,968**
	Thembisa	20% (*n* = 8,847)	45% (*n* = 20,467)	35% (*n* = 15,872)	**45,186**

In Zimbabwe, differences between models were generally historical; models mostly aligned well with regard to future projections. Optima HIV did project a lower absolute number of HIV-related deaths than the other models; in 2025 this was 3,666 (compared to the maximum estimate of 17,475 from EMOD). More variation was seen in Malawi; TLO estimates were highest over time (17,835 in 2025) and Optima HIV estimates were lowest (4,580 in 2025). In South Africa, three out of four models showed similar trends over time, forecasting declines of varying magnitudes in the total number of HIV-related deaths from around 2007. In contrast with other models, the number of HIV-related deaths in EMOD declined until 2022 before rising, plateauing at 150,000 by 2036.

### Deaths along the HIV care cascade in 2025

**Fig 3:**
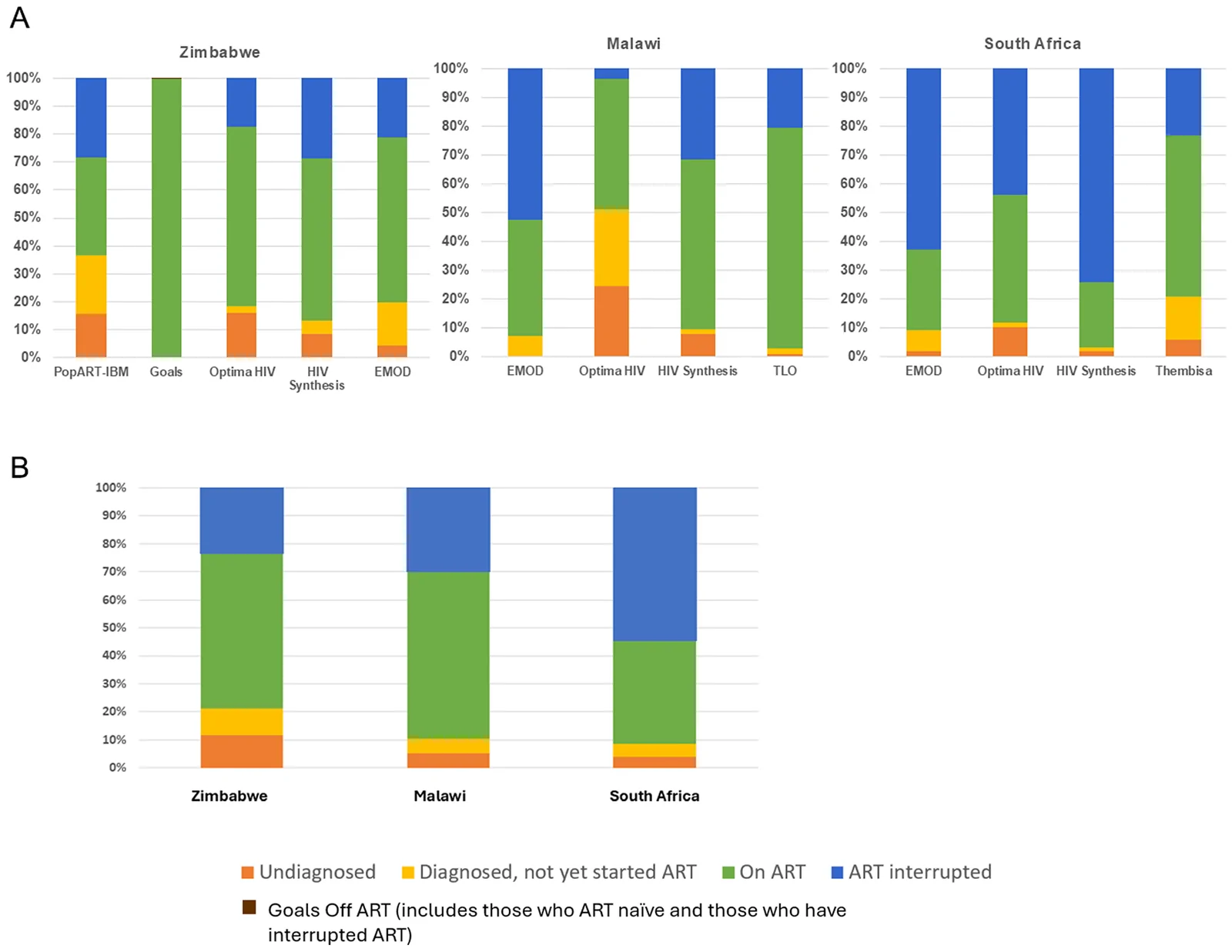
Proportion of HIV-related deaths in 2025. **(A)** Proportion of HIV-related deaths occurring amongst those undiagnosed, diagnosed without having started ART, on ART and those who have interrupted ART in Zimbabwe, Malawi and South Africa in 2025. **(B)** Median proportion of HIV-related deaths across models occurring amongst those undiagnosed, diagnosed without having started ART, on ART and those who have interrupted ART in Zimbabwe, Malawi and South Africa in 2025. x-axis: model; y-axis: percentage.

In Zimbabwe in 2025, all models agreed that people on ART accounted for the largest share of HIV-related deaths, though the proportions varied (Fig 3A).

Among models reporting all four care cascade stages, deaths among those on ART ranged from 35% (PopART-IBM) to 64% (Optima HIV). In Goals, where HIV-related deaths were stratified as either on ART or not on ART, nearly all HIV-related deaths were attributed to people on ART. HIV Synthesis, PopART-IBM, and EMOD estimated that over 90% of ART-related deaths occurred in individuals with viral non-suppression and CD4 <200/mm^3^, compared with lower proportions in Optima HIV (35% and 50%, respectively). The second-largest share of HIV-related deaths according to all models was among those who had interrupted treatment, ranging from 17% (Optima HIV) to 29% (HIV Synthesis). HIV-related deaths among undiagnosed individuals ranged from 4% (EMOD) to 16% (Optima HIV). Deaths among those diagnosed but not yet on ART were low in Optima HIV and HIV Synthesis (<5%) but higher in PopART-IBM (21%) and EMOD (15%).

In Malawi, the distribution of HIV-related deaths across care stages was similar to Zimbabwe (Fig 3A). Most deaths were among those on ART (40% in EMOD to 76% in TLO) and those who had interrupted treatment (21% in TLO to 53% in EMOD). Among people on ART, >75% of deaths occurred in those with viral non-suppression in HIV Synthesis and EMOD, compared with lower levels in TLO (57%) and Optima HIV (34%). HIV Synthesis and EMOD also estimated that >85% of ART-related deaths occurred in individuals with CD4 <200, versus 69% in TLO and 56% in Optima HIV. HIV-related deaths among undiagnosed people or those diagnosed but not yet on ART were low in most models, though Optima HIV estimated higher proportions (25% and 27%, respectively).

In South Africa, a high proportion of HIV-related deaths occurred among people who had interrupted ART (23% in Thembisa to 74% in HIV Synthesis) (Fig 3A). Of note, Thembisa outputs reflected lower bounds for those on ART and upper bounds for those who had interrupted ART. HIV-related deaths among people currently on ART ranged from 23% (HIV Synthesis) to 56% (Thembisa), with viral load and CD4 patterns similar to Zimbabwe and Malawi. Around 20% of deaths occurred among those undiagnosed or diagnosed but not yet on ART in Thembisa, compared with roughly 10% in the other models.

Fig 3B shows the median number of deaths across models for each setting.

### Trends over time

**Fig 4:**
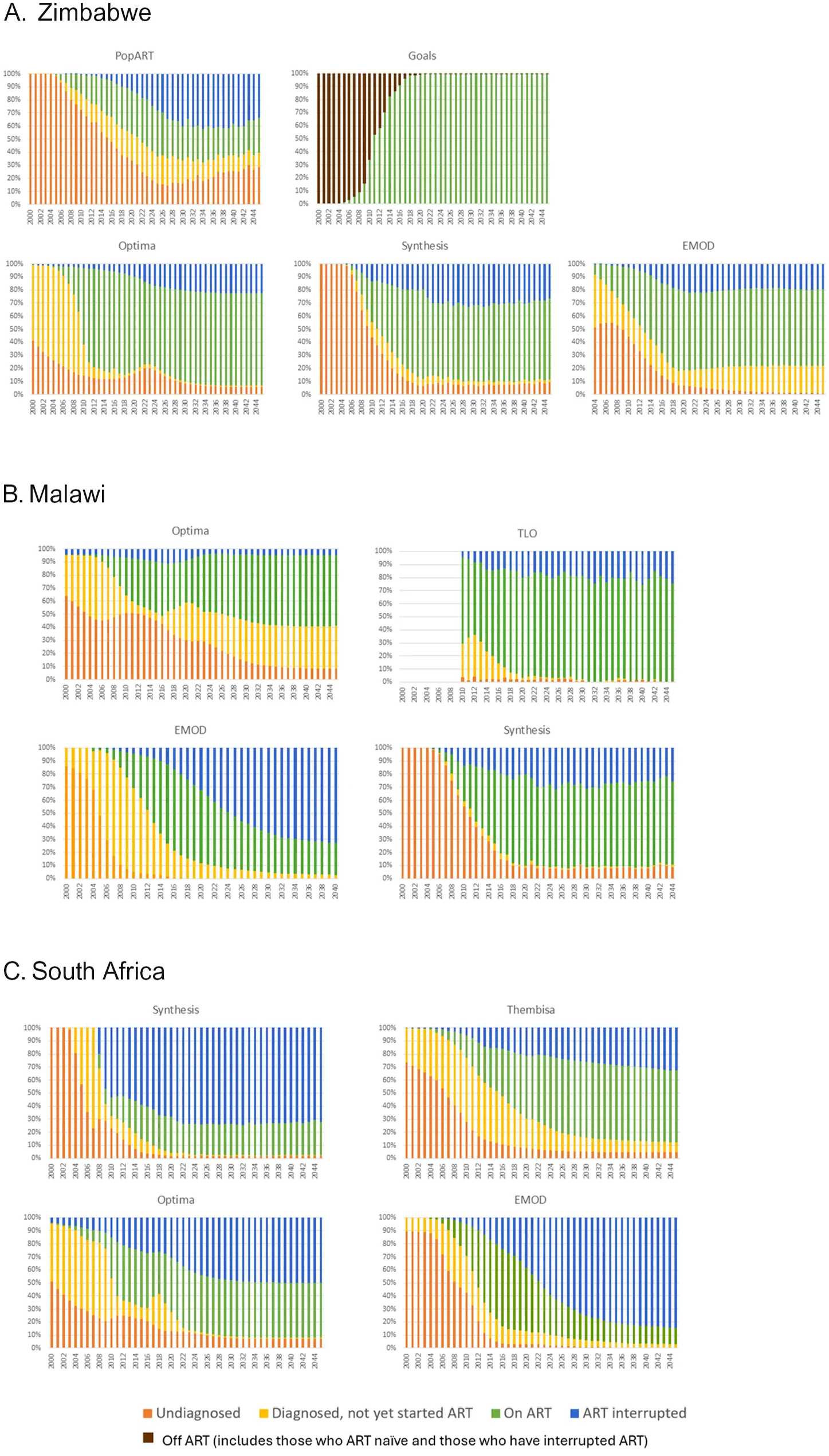
Proportion of HIV-related deaths occurring amongst those undiagnosed, diagnosed without having started ART, on ART and those who have interrupted ART in Zimbabwe, Malawi and South Africa from 2000 to 2045. x-axis: year; y-axis: percentage.

In Zimbabwe, the proportion of HIV-related deaths amongst those undiagnosed people fell considerably after 2000 and stayed below 20% in future projections for Optima HIV, HIV Synthesis, and EMOD (Fig 4A).

PopART-IBM, however, projected an increase after 2025, reaching nearly 30% by 2045. HIV-related deaths among those diagnosed but not yet on ART declined from 2000 to around 2020 in Optima HIV, HIV Synthesis, and EMOD, but future trends differed: Optima HIV and HIV Synthesis stabilised below 5% from 2025, while EMOD projected an increase, stabilising at 20% from 2030. PopART-IBM showed an increase to 21% by 2025, then a decline to 11% by 2045. HIV-related deaths among those on ART remained relatively stable over time across models, though at different magnitudes. HIV-related deaths occurring during ART interruption increased gradually over time before plateauing at 20% (Optima HIV, EMOD) to 40% (PopART-IBM). Sex and age differences were minimal (Figs B and E in S1 Appendix).

In all models of Malawi, the proportion of HIV-related deaths among undiagnosed people fell considerably after 2000 (Fig 4B). Two models projected it would stabilise at ~10% over the next 20 years, while the other two projected it would fall to near zero. Similar dichotomy was projected for deaths among those diagnosed but not yet on ART: three models showed a steady decline to below 5%, while Optima HIV projected an increase after 2016, reaching 30% by 2025. The proportion of HIV-related deaths among those on ART increased over time and plateaued around 2020 in three models but declined after 2020 in EMOD. HIV-related deaths among those who had interrupted treatment also varied, rising in EMOD (from 52% in 2025 to 72% in 2045) but remaining relatively stable in the other models (4% in Optima HIV to 31% in HIV Synthesis). Again differences between age and sex were minimal (Figs C and E in S1 Appendix).

In South Africa, trends differed from Malawi and Zimbabwe but were generally consistent across models (Fig 4C). The proportion of HIV-related deaths among undiagnosed people dropped to low levels after 2000 and remained below 10% from 2025 onward. Similar patterns were seen for those diagnosed but not yet on ART, ranging from 1% (Optima HIV, Synthesis) to 15% (Thembisa). The proportion of HIV-related deaths among people on ART stayed stable in Optima HIV and HIV Synthesis (about 20%–55% from 2025), while EMOD projected a decline from 28% in 2025 to 13% in 2045. HIV-related deaths among those who had interrupted ART were higher than in Zimbabwe or Malawi. Two models showed stable levels from 2025 (45% in Optima HIV to 75% in HIV Synthesis). EMOD and Thembisa projected increases: from 63% and 23% in 2025 to 84% and 32% by 2045.

Differences by age and sex are shown in Figs D and E in S1 Appendix.

### Time since starting ART, viral load and CD4 count at time of death

The majority of HIV-related deaths across settings and models occurred amongst people who had started ART over six months ago (Fig E in S1 Appendix); in Zimbabwe this was >80% across age groups according to HIV Synthesis and Goals, and >70% in those over 35 according to PopART-IBM and EMOD (with slightly lower percentages amongst those aged under 35). Similar trends were seen in Malawi and South Africa.

In Zimbabwe, over 60% of HIV-related deaths occurred amongst people with an undetectable viral load according to Optima HIV, with slight variations according to age (Fig F in S1 Appendix). This proportion was considerably lower according to HIV Synthesis (<10% for those aged 15–49, 40% for those aged 50+) and according to PopART-IBM and EMOD, no HIV-related deaths occurred amongst those with an undetectable viral load. GOALS did not report this outcome. Similar patterns were seen with the Malawi and South Africa projections according to Optima HIV and HIV Synthesis. No HIV-related deaths occurred amongst those with an undetectable viral load according to EMOD in any setting, while in Malawi, according to TLO, around 40% of HIV-related deaths occurred amongst people with an undetectable viral load.

While there was some variation with age, in 2025 around 60% of HIV-related deaths occurred in people with CD4 <200 across settings according to Optima HIV and TLO (Fig G in S1 Appendix). This was considerably higher according to EMOD at >80%, and higher still according to HIV Synthesis and PopART-IBM at greater than 90%.

## Discussion

In this collaboration within the HIV Modelling Consortium, we compiled results from multiple models to investigate HIV-related mortality across different stages of the HIV care cascade in Zimbabwe, Malawi and South Africa. Models aligned well with respect to key HIV characteristics in each of the settings, albeit with some variation in the projected proportion of diagnosed PWH on ART and number of new HIV infections. There was considerable variation in the projected number of HIV-related deaths within specific settings, with models at the higher end of projections having 4-fold higher numbers compared to those at the lower end of projections. However, within each setting, models generally agreed on the answer to our primary research question of which category of the care cascade accounted for the most HIV-related deaths. In Zimbabwe and Malawi, most models indicated that the majority of HIV-related deaths occurred among people with HIV (PWH) who were receiving ART, whereas in South Africa, most models showed that HIV-related deaths were predominantly among those who had interrupted ART.

Variation in the projected proportion of diagnosed PWH on ART and number of new HIV infections has been seen in previous publications of this group and is likely explained by the varying underlying structures and assumptions of specific models [15]. The variation in the number of HIV-related deaths is partially due to different assumptions over future changes in testing and ART uptake and retention but also due to differing definitions of ‘HIV-related’ and factors impacting likelihood of HIV-related deaths (Table A in S1 Appendix). For example, models that have higher rates of mortality while on ART would probably tend to estimate a relatively high absolute number of HIV-related deaths. Further, Optima HIV used a stricter definition of ‘HIV-related deaths’ compared to the other models, resulting in a lower projected number of total HIV-related deaths. The difference between the number of total HIV-related deaths was most pronounced among those aged 50 and over, where the majority of deaths in Optima HIV were attributed to general “all cause” factors rather than specific HIV-related causes. In EMOD, HIV Synthesis and Thembisa, in the absence of treatment, older people were assumed to progress to advanced stages of HIV much more quickly than younger individuals, resulting in a higher number of HIV-related deaths than in Optima HIV.

In addition to differences in assumptions about the future course of the epidemic, variation in model structure is also likely to contribute to the differences in predicted absolute numbers of HIV-related deaths observed. These analyses included both individual-based models and compartmental models, which represent disease progression, treatment dynamics, and mortality processes in different ways. These structural differences can influence how individuals move through the HIV care cascade and how deaths are generated within the model, including the interpretation of HIV-related deaths as distinct from other classifications; for example, AIDS-related deaths or excess deaths among people living with HIV, and therefore may contribute to variation in the projected number of deaths. Some models may include a larger proportion of the HIV population in higher-mortality strata such as individuals who have interrupted treatment. These differences in underlying population structure and assumptions about treatment engagement could contribute to the observed variation in mortality outcomes across models. Long-term trends, however, remained consistent across models and settings, with most projections indicating a plateau or decline in the absolute number of HIV-related deaths compared to earlier levels. The exception to this was the EMOD projections for South Africa, where an upward trend was observed. This increase was primarily attributed to population growth, suggesting that the rise in HIV deaths was largely driven by demographic changes rather than a worsening epidemic.

A general challenge in HIV modelling is the reliance on all-cause mortality estimates in PWH. Given the limited death certification in the region, even all-cause mortality estimates are difficult to ascertain, but it is particularly difficult to ascertain from these the actual HIV-related mortality estimates. Most models estimate the HIV-related mortality rate by subtracting the background (HIV–negative) mortality rate from the all-cause mortality rate in PWH, but this method can be inaccurate. For example, in high-income settings, Trickey and colleagues found that around half of excess mortality in PWH was in fact non-AIDS-related, which is not surprising given the high ART coverage [33]. This suggests that comorbidities (while potentially confounded by other factors such as smoking) are a major cause of death amongst PWH, highlighting the importance of effectively managing these conditions alongside HIV.

In Zimbabwe and Malawi, in line with the high proportion of people diagnosed with HIV, and of those diagnosed, on ART, most models found that the highest proportion of HIV-related deaths occurred amongst PWH on ART. There were some exceptions to this; PopART-IBM modelling Zimbabwe and EMOD modelling Malawi, had the highest proportion of HIV-related deaths occurring amongst those who died after having interrupted ART. In Zimbabwe, Optima HIV, HIV Synthesis and EMOD showed that those who had interrupted ART made up the second largest category of HIV-related deaths. Conversely, Goals had no HIV-related deaths occurring amongst those off ART after 2022. This is likely explained by differing assumptions, for example, in the Goals model, those with low CD4 counts are more likely to initiate ART and given the high ART coverage, most PWH would therefore be on ART. In HIV Synthesis, CD4 count does not influence the chances of starting or interrupting ART (since it is generally assumed to be not measured with the exception of a single value at the time of entry into/return to care) and hence those with the lowest CD4 nadir are not necessarily those on ART (though other assumptions around disease stage do result in some PWH who are severely ill being tested for HIV). Further, Goals does not distinguish those off ART by those undiagnosed or diagnosed without having started ART; in the other models, these latter two categories account for at least 10% of all HIV-related deaths. In Malawi, from 2020 onwards, the second highest category to which HIV-related deaths are attributable was also those off ART—though whether this was amongst those diagnosed but had not started ART, or amongst those who had interrupted ART was dependent on the model; Optima HIV projected the former, while TLO and HIV Synthesis projected the latter.

In South Africa, in line with the lower percentage of PWH on ART (81% [13]), deaths amongst those who had interrupted ART accounted for the majority of HIV-related deaths according to most models, followed by deaths amongst those on ART. Interruption rates in South Africa are high, reasons for which include stigma, mobility issues and inability to prioritise appointments due to other challenges [34]. While most models agreed that the proportion of HIV-related deaths was higher amongst those interrupting treatment, the magnitude of these proportions varied, likely due to differing assumptions on retention rates. In practice, in all settings, PWH who are considered lost to follow-up may resume ART (perhaps temporarily) at another facility, but without unique patient identifiers and electronic records, it is not possible to link these patients and hence data is sparse. Models differed in their approach to adjusting for potential biases due to loss to follow-up (LTFU); some assumed individuals lost to follow-up had interrupted treatment, while others did not. It is also possible that some deaths were classified as having occurred when ART was interrupted but actually occurred amongst people on ART with low adherence.

Despite any potential misclassification, HIV-related deaths amongst those on ART accounted for the second highest category of the care cascade in South Africa. In high-income settings, it is well evidenced that ART has dramatically improved life expectancy and that mortality while on ART has considerably declined over the last 30 years [33]. However, this is not necessarily true of African settings after controlling for changes over time in baseline characteristics and treatment duration, particularly in the first 12 months after ART initiation [35]. Sub-optimal adherence is likely a strong explanation as to why a relatively high proportion of deaths are attributable to people on ART. This is further evidenced by our study showing that most HIV-related deaths occurring amongst those on ART did so after 6 months of ART initiation. However, we did also see that in some models in those aged under 35, nearly 40% of HIV-related deaths amongst those on ART occurred within 6 months of starting ART. While ART duration is likely to be longer in older PWH, this may also suggest that those of younger, perhaps working age, are presenting far too late for care. Similar findings were seen in Tanzania, although the median age of the population was older at 35 years; those on ART but presenting late with low CD4 counts or WHO 3/4 conditions were at increased risk of mortality [36].

Differences between models may partly be explained by calibration processes. Modelling groups were not required to align their calibration approaches or the specific data points used. However, modelling groups have previously collaborated on several projects and, for both this and earlier work, discussed instances where calibration approaches and baseline outputs differed and the reasons for these differences. In particular, most of the modelling groups collaborated to assess the impact of ART disruptions during COVID-19 [14]. All groups consistently found significant increases in HIV-related deaths if ART were stopped, indicating a strong consensus in capturing the effects of ART interruptions. In these analyses, models were broadly consistent with the available data points (Fig 1). Nevertheless, variation in baseline cascade indicators may explain differences in HIV-related death categories. For example, in Zimbabwe, Pop-ART IBM estimated a slightly lower proportion of diagnosed individuals on ART, and projected a higher proportion of HIV-related deaths occurring among those diagnosed but not on ART and among those who had interrupted ART compared to the other models. Similarly, in Malawi, Optima estimated a lower proportion of individuals diagnosed and on ART compared with the other models, which may explain the higher proportion of deaths occurring among individuals who were diagnosed but had not initiated ART.

As seen already in Zimbabwe and Malawi, as countries get closer to achieving the 95-95-95 HIV testing, treatment, and viral suppression targets, the proportion of HIV-related deaths amongst PWH on ART is likely to increase. Adherence counselling will be key in ensuring that those with unsuppressed viral loads are back on target to achieving an undetectable viral load [37]. In settings such as South Africa where interruption rates are high, interventions focussing on retention are needed. These might, for example, include differentiated service delivery models [38], easier access to treatment [39] and focus on re-engagement in care [40].

Modelling studies are limited by the availability of empirical data. In Africa, the majority of deaths are not recorded in a national registry and annual death records are only available for a third of countries in the region [3]. Even when there is a high completeness of vital registration, a substantial proportion of HIV-related deaths are incorrectly attributed to other causes [41]. It is therefore very unlikely that HIV-related deaths are well recorded. This explains the wide range of absolute number of HIV-related deaths estimated by different models in this study. In order to measure success of interventions, not only for HIV, but across healthcare, governments must allocate budgets to building and maintaining death registries. This is also crucial in measuring progress towards the Sustainable Development Goals, which include targets around mortality. Models did also differ in their definition of ‘HIV-related’ deaths, with different model classifications especially for older populations reflecting practical and clinical challenges in the attribution of excess deaths among people living with HIV. By presenting proportions, however, we were still able to compare meaningfully across models. Finally, while models that were able to did incorporate uncertainty around parameter estimates, we elected not to present uncertainty bounds due to the volume of data presented; presenting such bounds in the Figures would have made readability difficult. However, the structural uncertainty presented by comparing several models in each setting gives a good, if not better, idea of uncertainty around the estimates presented; further, structural uncertainty is not usually possible to ascertain in single-model analysis.

Multi-model analyses provide a considerably more robust perspective than any single-model approach. When results are consistent, they provide strong evidence to public health and policymakers and when there are discrepancies, teams revisited their assumptions to understand and explain the differences. This collaborative process represents a substantial effort by each modelling group, far exceeding what would be required to produce a paper based on a single model.

From a public health perspective, it is important for policymakers to interpret results in the context of the uncertainty surrounding them. This does not require understanding the technical details of each model’s assumptions, but rather an appreciation that no model is perfect and estimates can vary, as we have explained throughout this Discussion. In this multi-model comparison, most models found that the majority of HIV-related deaths in Zimbabwe and Malawi occurred amongst those on ART, followed by those who had interrupted ART. This suggests, assuming ART access and testing remains unaffected, that efforts need to be focussed on adherence counselling and retention in these settings. In South Africa, most models agreed that deaths among individuals who had interrupted ART represented the largest proportion, highlighting the need to prioritise efforts to re-engage people in care in this setting.

The HIV funding landscape is in crisis. Gains made over the last 20 years with the introduction of ART and successful HIV programmes in Africa are at risk of being reversed. We have shown here that HIV-related deaths are still a major cause for concern in the region. Notably, these analyses were conducted before the recent funding changes and at the time this discussion was written, there was insufficient programme data to determine the actual or likely impact of the cuts, particularly with respect to mortality outcomes. Hence, the situation following the cuts may be even more concerning. While ART access has been prioritised thus far, ensuring its continued prioritisation is essential [41]. There is concern that interventions around adherence counselling and retention in care, which are necessary to ensure all those who have started ART are using it optimally, will be impacted. This may result in more people in the ‘interrupted ART’ category leading to further avoidable deaths. Further, those undiagnosed are at risk not only of premature death, but also of transmitting HIV to others, and hence interventions to test and treat are vital. Measuring the impact of such interventions and planning healthcare priorities for the future is, however, reliant on death registries; collecting data on deaths is key to improving healthcare across the region. Alongside such registries which can aid with identifying cause of death, modelling is vital to estimate more specific populations in which deaths occur such as those presented in this study, and for some populations (i.e., deaths amongst those undiagnosed) the only way of doing so. In combination, empirical data and modelling can best guide interventions to ensure HIV-related deaths continue to decline in the region.

## Supporting information

S1 Appendix Supplementary table and figures material. (DOCX)

## Abbreviations

ART: antiretroviral therapy
IeDEA: International epidemiology Databases to Evaluate AIDS
MIHPSA: Modelling to Inform HIV Programmes in sub-Saharan Africa
PWH: People with HIV
TLO: Thanzi La Onse
