# Supplementary material for: HIV diagnosis and treatment status in individuals dying from HIV in Zimbabwe, Malawi, and South Africa: A model comparison analysis
Source: PLoS Med. 2026 Sep 10;23(9):e1004864. doi: 10.1371/journal.pmed.1004864 (PMC13561354; doi:10.1371/journal.pmed.1004864)
Supplement: Supplementary file 1 [file pmed.1004864.s001.docx]

**Supplementary material**

Table A: Key characteristics of the contributing models

|  | **Goals** | **Optima HIV** | **HIV Synthesis** | **EMOD** | **Thembisa** | **PopART-IBM** | **TLO** |
| --- | --- | --- | --- | --- | --- | --- | --- |
| Modelled countries | **Zimbabwe** | **Zimbabwe, Malawi,**  **South Africa** | **Zimbabwe, Malawi,**  **South Africa** | **Malawi,**  **South Africa** | **South Africa** | **Zimbabwe** | **Malawi** |
| **Structure** | Compartmental model with disaggregation by sex and risk group. 1-year time step | Compartmental model with population disaggregation depending on setting, including sex, age, and risk group. 2.4-month time step. | Individual-based stochastic model. 3-month time step. | Network individual-based stochastic model. Monthly time step. | Compartmental model with disaggregation by sex, age, risk group and marital status. 1-month time step. | Individual-based stochastic model with dynamic sexual network, timestep 1/48 year | Individual-based, dynamic simulation model of the whole health system. Captures disease transmission, natural history, healthcare access, and resource constraints. Modular design allows integration of multiple diseases and interventions. Includes demographics, care-seeking, and health system interactions. |
| **Modelling HIV-related deaths** | Mortality rates for PLHIV not on ART are specified by CD4 count and age. For those on ART mortality rates are specified by sex, age, CD4 count at ART initiation, duration on ART and geographic region. | Mortality both on ART and off ART depends on current CD4 count and ART status (un/suppressive). | Base rate of clinical disease leading to death is dependent on CD4 count, viral load, age and being on ART. For those with TB/WHO 3/WHO 4, the base rate is multiplied by a given factor. | Risk of mortality while on ART is determined by age, sex, CD4 count at treatment initiation and duration on treatment. | HIV mortality rates in ART-naïve individuals depends on age, sex and CD4, and can change over time. Mortality rates in ART-experienced individuals depend on age, sex, baseline CD4 and time since ART initiation. | Risk of AIDS-related mortality while on ART is zero if virally suppressed, and only occurs for individuals not on ART or on ART but not virally suppressed when CD4<200, for which it is dependent on set-point viral load and sex. | HIV-related mortality arises from progression through disease stages in untreated individuals or from treatment failure. The model simulates individual-level ART initiation, adherence, and retention. Death risk is determined by disease stage, viral suppression status, and co-infections. |
| **Approach to calibration to data and estimates for specific settings** | Epidemiological parameters (probability of transmission per act; variation by stage of infection, presence of other STIs, effectiveness of condoms and ART) are varied to fit the model to prevalence estimates from surveillance and surveys. | Epidemiological parameters (probability of transmission per act, variation by stage of infection (informed by CD4 counts and viral load monitoring), HIV testing rate, mortality rate, presence of other ulcerative STIs and/or tuberculosis, as well as effectiveness of condoms, circumcision, and un/suppressive ART) are varied to fit the model to prevalence estimates from surveillance and surveys. | Parameters relating to population characteristics, sexual behaviour (condomless sex) and age-gender mixing, HIV acquisition, HIV testing, natural history (CD4 count and viral load), ART, risk of AIDS and death are varied within plausible bounds to create a range of setting scenarios amongst which we select ones with outputs corresponding to observed data. | The model is parameterized with demographic data, including population size, fertility, mortality, as well as age- and sex-specific epidemiological data, including prevalence, incidence, ART coverage, and voluntary male circumcision coverage. Calibration was performed using a parallel simultaneous perturbation optimization (PSPO) algorithm. Roulette resampling in proportion to the likelihood of each simulation was used to select 250 model parameter sets. | HIV transmission rates per sex act, HIV survival parameters, sexual behavior parameters, HIV testing rates and ART initiation rates are varied in calibrating the model to South African HIV prevalence data (antenatal, household and key population surveys), mortality data, HIV testing history data, ART programme data and paediatric HIV data, using a Bayesian approach. | The model is parameterized using age-and sex- specific data on sexual partnerships, and HIV prevention, testing and treatment uptake from a population cohort in Manicaland, Zimbabwe. Demographic data, including population size, fertility and mortality come from national estimates. The model is calibrated as follows. Firstly Latin hypercube sampling is used to generate N=100,000 parameter sets. The resulting runs are then target-fitted to national data on HIV prevalence for Zimbabwe at four time-points stratified by sex, producing 10 model parameter sets consistent with all data. | Calibrated using national and subnational data on demography, disease prevalence/incidence, service coverage, and mortality. Multiple calibration approaches are used depending on the model component and data availability. These include empirical parameter grid search, Bayesian calibration and rejection sampling methods. Parameters are adjusted to fit routine surveillance data and household survey indicators. |
| **Sexual behaviour** | Behaviours (number of partners, acts per partner, condoms use, needle sharing) differ by risk group: female sex workers, male clients, males and females with non-regular partners, faithful couples, MSM and PWID. | Behaviours (type of partners (regular, casual, or commercial sexual; injecting), acts per partner, condom use and needle sharing differ by age and risk group (female sex workers, clients of sex workers, MSM, and PWID). | Two types of condomless sexual partnerships (long- and short-term). Long-term partnerships are remembered over time. Rates of condomless sex contact depends on age, type of partnership and gender. MSM and PWID are not explicitly modelled. | Four types of sexual partnerships (marital, informal, transitory, and commercial) are formed according to specifiable partner age patterns and tracked over time. | Three types of sexual partnership (marital/cohabiting, short-term, commercial), with different coital frequencies and condom use for each. Rates of short-term and commercial sex contact depend on risk group (high/low), age, marital status, and sex. Separate assumptions for MSM. | Two types of sexual partnerships (casual and long-term), each with their own specific probability of condom use. The population is subdivided into three risk groups, with higher risk individuals having higher rates of partner acquisition. Partnerships are formed based on risk- and age-specific partnership formation rates and an age-mixing matrix (by sex) derived from Manicaland general population survey. | Sexual partnerships are not explicitly modelled. Adult HIV transmission is simulated probabilistically, with the probability of infection determined by a calibrated transmission rate, sex-specific HIV prevalence in the opposite sex, and a set of individual-level relative risks (demographic and behavioural characteristics including age, sex, residence (urban/rural), wealth, education, circumcision status, exposure to behaviour change interventions, and PrEP).  Risk for women is proportional to the prevalence of non-virally suppressed HIV among men, and vice versa. An additional risk multiplier is applied for female sex workers. |
| **Changes to sexual behaviour after diagnosis, starting ART** | No changes to sexual behavior as a result of diagnosis or starting ART. | No changes to sexual behaviour assumed as a result of diagnosis or starting ART. | Reduction in condomless sex after diagnosis  No change resulting from ART initiation to viral suppression | No changes to sexual behaviour assumed as a result of diagnosis or starting ART. | Increased frequency of sexual activity (due to increased CD4) and higher levels of condom usage. | No changes to sexual behaviour assumed as a result of diagnosis or starting ART. | Behaviour change post-diagnosis or ART is not explicitly modelled. Reduced transmission risk is captured via viral suppression (zero transmission if suppressed). |
| **HIV acquisition determinants (including prevention interventions included)** | Acquisition risk depends on characteristics of the individual (number of partners, circumcision status (males), PrEP use), the partner population (HIV prevalence, ART use, stage of infection), and the partnerships (acts per partner, prevalence of other STIs, type of act, condom use). | Acquisition risk depends on characteristics of the individual (number of partners, number of injections), and the partnerships (type of partner, acts per partner, condom use, circumcision status (males), PMTCT status, PrEP and PEP use, opiate substitution therapy use, receptive needle sharing), and HIV status by population (HIV testing, HIV diagnosis, HIV prevalence, un/suppressive ART use, stage of infection). | Acquisition risk depends on characteristics of the individual (age, sex, number of condomless sex partner, circumcision status (males), PrEP use), the partner population (HIV prevalence, viral load) and the partnership (type of condomless sex partnership).  Transmission of virus with specific drug resistance mutations is modelled. | Acquisition risk depends on characteristics of the individual (number of partners, circumcision status (males), condom use, other STIs and acts per partner) and of the partner (HIV prevalence, ART use, and stage of infection.) | Acquisition risk per sex act depends on characteristics of the individual (age, sex, risk group, type of relationship, circumcision status (males), and PrEP use) and HIV+ partner characteristics (condom use, acute infection, CD4 count, ART status, viral suppression). | Acquisition occurs only in serodiscordant partnerships. HIV acquisition can occur at each timestep in a serodiscordant partnership (1/48 year) and depends on whether condoms are used in the partnership, whether the negative individual is on PrEP (and circumcised, if male), whether the positive partner is on ART, the stage of infection (acute, or CD4 stage if chronic) and set-point viral load of the HIV-positive partner. | Acquisition risk varies by sex, age, residence, wealth, education, circumcision, and PrEP (if included). Increased risk applied for female sex workers. |
| **HIV natural history** | Rate of decline of CD4 count off ART depends on current CD4 count and age. Mortality off ART depends on age, and CD4 count. | Rate of decline of CD4 count and viral load off ART depends on current CD4 count and viral load. Mortality off ART depends on current CD4 count | Rate of decline of CD4 count dependent on current viral load. Viral load increases over time. Risk of AIDS and death dependent on current CD4 count, current viral load, age, current use of co-trimoxazole. | HIV prognosis is calculated using a Weibull distribution where the parameters of the distribution are derived from CD4 count and age at the time of infection. CD4 count decreases from the time of infection while off ART. | After acute infection phase, individual progresses through four CD4 stages in absence of ART, with rates of CD4 progression depending on age and sex. | After acute infection phase, individuals not on ART progress through four CD4 stages, with rates of CD4 progression depending on the set point viral load of the individual. Untreated AIDS-related mortality occurs when CD4<200. | Simulates disease stages based on time since infection and symptom severity. Key mechanistic links include increased susceptibility and progression rates for TB, malaria, ALRI and other diseases due to HIV-induced immunosuppression, which are explicitly incorporated to capture co-infection dynamics and their impact on morbidity and mortality. Mortality risk increases with HIV stage. |
| **HIV testing / diagnosis** | Testing is by modality and population group and determines knowledge of status, but this is not linked to transmission since ART coverage is direct input. | Testing is modelled by modality, population group, and year, which determines knowledge of status and allows linkage to care and initiation on ART based on treatment coverage level. | Whether a person is tested or not is defined in each period. Testing is indicated in antenatal clinic, for symptoms potentially of HIV, and potentially every 6 months for FSW. Also, general testing with various degrees of targeting at people with higher probability of infection and dependent on testing history. | HIV testing/diagnosis occurs voluntarily, at antenatal visits, or once symptomatic | Five types of testing in adults: antenatal, testing in patients with opportunistic infections, general testing, self-testing and index testing (following referral by an HIV-positive partner). General testing rates vary by age, sex, and testing history. | Testing is either general testing or symptomatic testing. General testing rates vary by sex and age, and over time, and are capped to not exceed 3 million tests per year from 2021 onwards. | Diagnosis occurs via time-varying testing rates by age/sex. Multiple testing modalities included, ANC, VCT, PITC, self-testing. Testing rates calibrated to the numbers of tests performed per year along with the ART coverage. Eligible individuals can initiate ART post-diagnosis. |
| **ART** | Includes initiation, suppression, dropout, and re-engagement pathways. | Includes initiation, suppression, dropout, and re-engagement pathways. | Includes initiation, suppression, dropout, and re-engagement pathways.  Specific drugs and their current level of activity given drug resistance, and current ART adherence determine viral load, CD4 count change and risk of further resistance | Includes initiation, suppression, dropout, and re-engagement pathways. | Includes initiation, suppression, dropout, and re-engagement pathways. | Includes initiation, suppression, dropout, and re-engagement pathways.  Individuals on ART and virally suppressed do not experience further disease progression or AIDS-related mortality. Those on ART but unsuppressed progress at a reduced rate 0.5-1 times that of those unsuppressed. | Includes initiation, suppression, dropout, and re-engagement pathways. |
| **ART interruption** | Return to CD4 count one category higher than at time of treatment initiation. Survival progression identical to those who are treatment naïve. | CD4 count decreases following dropout from ART. Prognosis is recalculated based on the age and CD4 count at the time of ART dropout. Computation of ART prognosis after re-enrolment on treatment is the same as the initial enrolment with prognosis parameters corresponding to age and CD4 count at re-enrolment. | Immediate (within 3 months) viral load return to pre-ART level, substantial initial decline in CD4 count towards pre-ART nadir | CD4 count decreases following dropout from ART. Prognosis is recalculated based on the age and CD4 count at the time of dropping off ART. Computation of ART prognosis after re-enrolling is identical to the initial enrolment with prognosis parameters corresponding to the age and CD4 count at re-enrolment. | Immediate return of CD4 and viral load to levels at time of ART initiation, after an ART interruption. | HIV progression restarts at the CD4 count at time of treatment initiation, and with disease progression and AIDS-related mortality identical to those who are treatment naïve. Individuals can restart ART in future. | ART interruption is modelled as temporary cessation of ART with defined probabilities of stopping and re-initiation, affecting viral rebound and transmission; parameters are informed by cohort and programme data on retention and adherence. |
| **Assumptions on future projections** | Constant coverage of the population in need for all prevention and treatment interventions at 2024 levels. | Constant coverage of the population in need for all prevention and treatment interventions at 2024 levels. | Rates of starting prevention services, testing rates and ART starting and stopping rates remain fixed at 2024 levels | Rates of initiation of prevention services, HIV testing, and ART initiation and discontinuation were assumed to remain constant at 2024 levels. | Rates of starting prevention services, testing, linkage to ART, and ART interruption remain unchanged from 2024 levels. | Probabilities of prevention services, and ART initiation and discontinuation remain unchanged from 2024 levels. Annual number of HIV tests remains fixed at 2024 levels. | All service coverage indicators (prevention, testing and treatment) were kept at 2024 levels for future projections. |

Fig A: HIV-related death cascade by sex in Zimbabwe

Fig B: HIV-related death cascade by sex in Malawi

Fig C: HIV-related death cascade by sex in South Africa

Fig D: HIV-related death cascade by age

Zimbabwe	Malawi

South Africa

Fig E: Amongst those who were on ART, percentage of deaths stratified by time since start of ART: <6 months (6m), >6 months

Zimbabwe	Malawi

South Africa

Fig F: Percentage of deaths stratified by viral load at time of death

Zimbabwe	Malawi

South Africa

Fig G: Percentage of deaths stratified by CD4 at time of death
